# GEAR: A database of Genomic Elements Associated with drug Resistance

**DOI:** 10.1038/srep44085

**Published:** 2017-03-15

**Authors:** Yin-Ying Wang, Wei-Hua Chen, Pei-Pei Xiao, Wen-Bin Xie, Qibin Luo, Peer Bork, Xing-Ming Zhao

**Affiliations:** 1 Department of Computer Science and Technology, Tongji University, Shanghai 201804, China; 2 Department of Electronic Engineering, City University of Hong Kong, Kowloon 999077, Hong Kong; 3 Key Laboratory of Molecular Biophysics of the Ministry of Education, Hubei Key Laboratory of Bioinformatics and Molecular-imaging, Department of Bioinformatics and Systems Biology, College of Life Science and Technology, Huazhong University of Science and Technology (HUST), Wuhan, Hubei 430074, China; 4 Beijing Institute of Genomics, Chinese Academy of Sciences, Beijing, 100101, China; 5 European Molecular Biology Laboratory (EMBL), Heidelberg, 69117, Germany

## Abstract

Drug resistance is becoming a serious problem that leads to the failure of standard treatments, which is generally developed because of genetic mutations of certain molecules. Here, we present GEAR (A database of **G**enomic **E**lements **A**ssociated with drug **R**esistance) that aims to provide comprehensive information about genomic elements (including genes, single-nucleotide polymorphisms and microRNAs) that are responsible for drug resistance. Right now, GEAR contains 1631 associations between 201 human drugs and 758 genes, 106 associations between 29 human drugs and 66 miRNAs, and 44 associations between 17 human drugs and 22 SNPs. These relationships are firstly extracted from primary literature with text mining and then manually curated. The drug resistome deposited in GEAR provides insights into the genetic factors underlying drug resistance. In addition, new indications and potential drug combinations can be identified based on the resistome. The GEAR database can be freely accessed through http://gear.comp-sysbio.org.

The emergence of drug resistance in clinic leads to the failure of standard treatments, thereby making the treatments of diseases more complex and costly. Specifically, drug resistance is commonly found during anti-infective treatments, such as tuberculosis (TB), Human immunodeficiency virus (HIV), and malaria. For example, drug resistant TB is prevalent in patients around the world, and multi-drug resistant TB is reported to occur in about 18% of TB patients in 2010 according to World Health Organization (WHO). Recently, acquired resistance was also emerged in cancer cell which not only become resistance to the drugs originally used to treat them, but also cross-resistant to the other drugs^1^. Therefore, understanding the mechanism of drug resistance may help to improve the drug therapy.

In general, the resistance to targeted therapy is developed due to certain genetic mutations or alteration of expression. Recently, a large number of mutations have been identified that are responsible for drug resistance with the utilization of high-throughput sequencing^2^. For example, the expression of P-glycoprotein (P-gp), a MDR1 protein product and ABC transporter, is responsible for multidrug resistance to anti-cancer drugs^3^. Moreover, numerous pre-clinical studies have demonstrated that the mutation of topo-I is a key determinant of CPT-11 sensitivity^4^. Nowadays, a number of such genes have been identified, which are unfortunately scattered in literature. In literature, large efforts have been made to collect drug resistance related genes, and some valuable resources have been constructed. For example, the HIV drug resistance database^5^,^6^, tuberculosis drug resistance mutation database^7^, antibiotic resistance database^8^,^9^, and cancer drug resistance database^10^, among others. Despite the great usefulness of those databases, most of them focus on specific drugs. For instance, the antibiotic resistance database (ARDB) mainly reports the mutations of bacteria genes that lead to resistance to antibiotics. The cancer drug resistance database (CancerDR) provides information on gene mutations that lead to resistance, where the associations were inferred based drug responses in cell lines without explicit evidence. Furthermore, most of existing databases provide gene mutations that are responsible for drug resistance. However, except for genes, those mutations outside of coding genes and within noncoding RNAs (e.g. miRNAs) may also cause drug resistance, which should be further considered^11^. Therefore, it is highly demanded to develop a comprehensive database about genetic factors conferring human drug resistances.

In this work, we present GEAR (A database of **G**enomic **E**lements **A**ssociated with drug **R**esistance) that aims to provide comprehensive information about genes, single-nucleotide polymorphisms (SNPs) and microRNAs (miRNAs) that are responsible for resistance to drugs. These associations between human drug resistance and genetic factors are firstly extracted from primary literature with text mining and are subsequently manually curated. Figure 1 presents the schematic content of GEAR. The GEAR database provides a user-friendly interactive interface, where cross-links and external links for drugs, genes, miRNAs and SNPs are also provided. Furthermore, the network visualization of the human drug resistome is also provided. The drug resistome deposited in GEAR provides insights into the genetic factors underlying drug resistance. In addition, new indications and potential drug combinations can be identified based on the resistome. The GEAR database can be freely accessed through http://gear.comp-sysbio.org.

## Results

### Database contents and implications

GEAR provides a user-friendly and powerful interface to query and visualize the data deposited in the database. The drugs can be easily queried with their PubChem IDs or Drug Names/Synonyms, whereas genes can be queried with their gene IDs or Gene Symbols/Synonyms. For each drug, its chemical structure, target proteins and therapeutic information can be found if available. For each gene, its functional annotations, pathway information and interaction partners can be easily retrieved. Similarly, detailed information about miRNAs and SNPs are also available in GEAR. Furthermore, the crosslinks between different molecules and external links to popular databases, e.g. PubChem and NCBI Gene^12^, are provided in GEAR. In addition, GEAR provides a very powerful and interactive visualization interface to the drug resistome consists of drug-gene/miRNA/SNP associations, which is visualized with Cytoscape plugins^13^. These networks can help to understand the molecular underpinnings of drug resistance in a systematic way.

### Distributions of drugs and resistant genes

By looking into the drug resistance events deposited in GEAR, we investigated the 201 drugs with known resistant genes. Based on the first level of the Anatomical Therapeutic Chemical (ATC) classification system^14^, these drugs can be grouped into 14 categories (as shown in Table 1). Among those drugs, we can see that anti-cancer and anti-infective agents are more likely to develop resistance as expected.

In addition, we investigated the functions of those genes that confer drug resistance by performing functional enrichment analysis on the resistant genes associated with drugs belonging to the same ATC category. The results on both pathway and Gene Ontology enrichment analysis imply that for each drug category, the resistant genes associated with those drugs tend to be enriched in biological processes related to drug effects (As shown in Supplementary Table I, where each sheet was represented by the first level of ATC codes). For example, it is known that the insensitivity to drug-induced apoptosis and the ejection of drugs by transporters are the common reasons for the resistance to anti-cancer drugs^3^. The functional enrichment analysis on the 635 resistant genes associated with cancer drugs indicate that these genes are enriched in the above mentioned resistant pathways and those related to drug therapies, e.g. ABC transporter, apoptosis, cell proliferation and P53 pathways. Furthermore, it is shown that the resistant genes associated with the same drug tend to interact with each other (*p*-*value* = 4.21e-161, *Fisher’s* exact test as shown in Supplementary Table II (F1)).

### Mechanisms of drug resistance unveiled by the resistome

**Figure 2:**
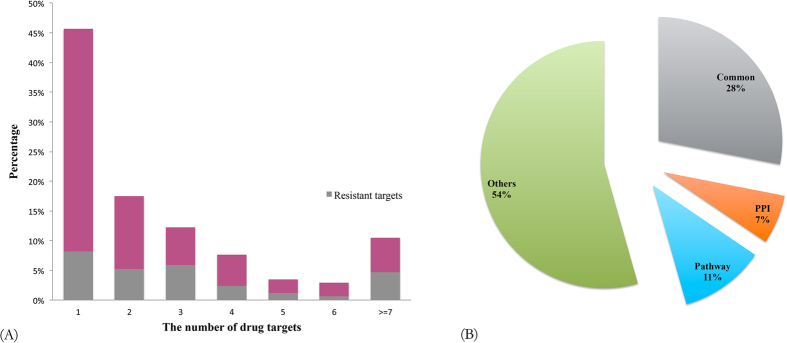
(**A**) The distribution of drugs according to the number of their targets, where the fraction of resistant genes in drug targets are also shown. (**B**) The distribution of drugs based on the relationships between their targets and associated resistant genes.

In general, the drug actions were accomplished by the interactions between drugs and their target proteins. Therefore, the target proteins are vital to understanding the mechanisms of drug actions. By investigating the targets of drugs (170 drugs have target information in GEAR), we found that almost half of the drugs (percentage = 46%) were single-target drugs and most of them targeted no more than 3 proteins as shown in Fig. 2A, implying that drugs with less targets are more likely to develop resistance. Indeed, we noticed that many of the targets of those drugs were also resistant genes. Therefore, it is not surprising that resistance tends to happen to drugs with less targets considering that diseases will bypass drug actions by mutating the drug targets when treated with single-target agents.

Recently, it has been found that except for the mutations to drug targets, the dysfunction of certain protein-protein interactions and pathways may also leads to the emergence of drug resistance^15^. According to the relationships between drug targets and their resistant genes, as shown in Fig. 2B, the drugs can be grouped into three categories: (a) drug targets are resistant genes; (b) drug targets interact with resistant genes; (c) drug targets and resistant genes belong to the same pathway(s). Figure 2B shows the distribution of the drugs with both target and resistant gene information. We found that among the 170 drugs with target information, there were 48 drugs whose target proteins were also resistant genes. For example, the patients who initially responded to Gefitinib eventually become resistant due to the somatic mutations in the EGFR gene that was targeted by Gefitinib^16^. A small part of drugs were found to develop resistance due to the interactions between their targets and resistant genes. The interactions between resistant genes and drug targets indicated that protein-protein interactions are promising drug targets as reported in literature^17^. For example, NFKB1 is one target of Dexamethasone while STAT3 was reported as a resistant gene of the drug in GEAR, where the drug was used to treat many inflammatory and autoimmune disorders. It has been reported that the interaction between NFKB1 and STAT3 plays a key role in the interaction between the malignant cell and its microenvironment, and promotes the development and progression of colon, gastric and liver cancers^18^. Therefore, it is reasonable to see the interaction between these two genes is associated with drug resistance.

Considering that drugs may develop resistance by blocking or rewiring the molecular contexts of drug targets, the pathway information about both drug targets and resistant genes can help to understand the molecular mechanisms underlying drug resistance. Among the drugs with both target and resistant gene information available, more than 10% of them have their targets and resistant genes in the same pathway(s). For instance, the resistant genes TP53, JUN and DLD of Etoposide participated in the JNK signaling pathway together with its target gene MAP2K7. The activation of the JNK pathway has been reported to promote the acquired resistance of T-cell acute lymphoblastic leukemia to Etoposide, implying the potential of JNK signaling pathway as a target in treating cancers^19^. Although the three types of relationships between drug targets and resistant genes can only explain half of resistant drugs, these findings can help better understand drug resistance and design more efficient drugs in the future.

### Drugs with same resistant genes tend to have similar therapies

It has been found that drugs with same targets tend to have similar mechanism and therapies. Here, we also investigated whether the drugs sharing resisted genes have similar therapies. By investigating the drugs that share resistant genes, we found that those drugs significantly tend to share the same ATC code (*p*-*value* = 9.48e-92, *Fisher’s* exact test as shown in Supplementary Table II (F2)) and therefore have similar therapeutic effects, where the first level of ATC codes was considered. In addition, we also found that the drug pairs sharing same resistant genes tend to have similar chemical structures (*p*-*value* = 3.36e-32, *Fisher’s* exact test as shown in Supplementary Table II (F2)), which further confirms that the drugs sharing resistant genes have similar therapies.

**Figure 3:**
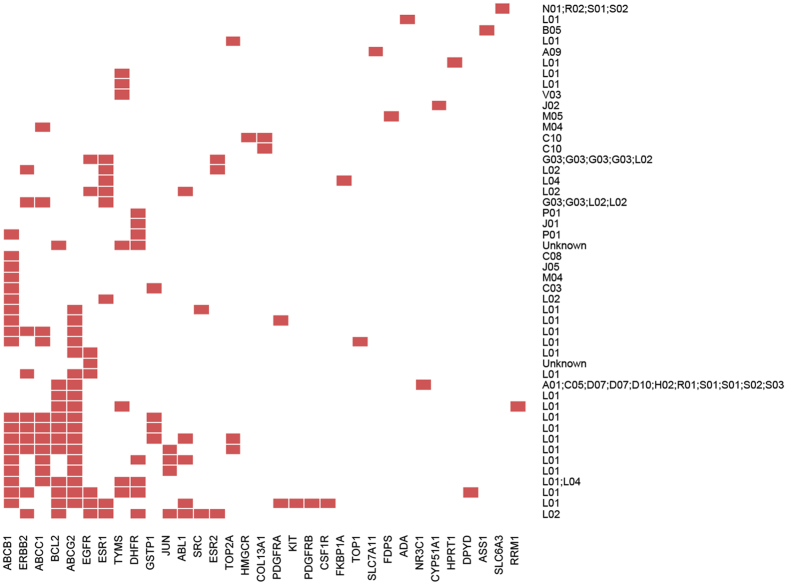
The clustering of drugs based on their resistant genes, where only resistant genes that are also targets of corresponding drugs are considered. Each row represents a drug denoted by the second level of ATC codes associated with the drug while each column denotes the resistant genes, and the red block means the association between a pair of drug and resistant gene while white ones mean no associations.

Figure 3 shows the clustering of those drugs whose resistant genes are also their targets. From the clustering results, we can see that drugs sharing resistant genes tend to have similar therapies. Furthermore, it can be seen that some genes are associated with multidrug resistance, especially the ATP-binding cassette (ABC) transporters that are responsible for decreased drug accumulation and the development of resistance to anticancer drugs. For example, the genes ABCB1, ABCG2 and ABCC1 were associated with multiple drugs and have been reported to be implicated in the efflux of anticancer drugs, such as Gefitinib, Docetaxel and Doxorubicin^20^. The results also showed that anticancer drugs (annotated with ATC code L01) may develop resistance with similar mechanisms, e.g. ABC transporters. Moreover, it was found that the resistance of drugs with endocrine therapy (annotated with ATC code L02) was associated with genes ESR1, ESR2 and ERBB2 that play important roles in hormone treatment. These findings indicated that similar drugs may have similar mechanisms when develop resistance.

## Expanded applications

### Resistome based drug repositioning

**Figure 4:**
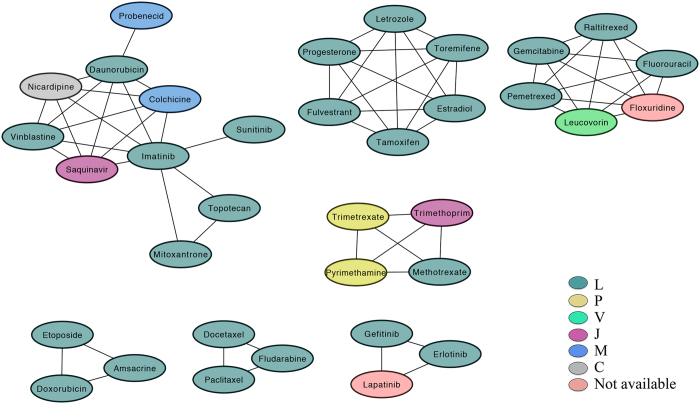
The modules from drug association network constructed based on the drug resistome from GEAR, where the nodes are drugs, the color of the nodes denote the first level of ATC codes and the linked drugs share at least one resistant gene.

From the findings observed above, we supposed that drugs sharing resistant genes may have similar therapies. Therefore, new indications are expected to be predicted based on drug resistant genes. With the drug resistome deposited in GEAR, a drug association network can be constructed where an edge will be laid between a pair of drugs if they share at least one resistant gene. In this way, a module extracted from the drug association network consists of drugs that share same resistant gene(s). Figure 4 shows the association network of drugs whose resistant genes are also target genes, where some modules can be clearly observed. By looking at the modules, we noticed that the drugs from the same module tend to share the same ATC codes (*p*-*value* = 2.24e-3 by *Fisher’s* exact test as shown in Supplementary Table II (F3)), where only the first level of the ATC code was considered here. In addition, we found that the drug pairs from the same module tend to have similar chemical structures (*p*-*value* = 2.23e-6 by *Fisher’s* exact test as shown in Supplementary Table II (F3)), whereas the similar chemical structure means similar therapy. Thus, by investigating the modules in the network, we can draw the conclusion that the drugs from the same module tend to have similar therapies, which can be used to predict new indications for old drugs. For example, the drugs Floxuridine and Lapatinib share resistant genes with anti-cancer agents while these two drugs have not been used for treating cancers. Based on the assumption that the drugs sharing resisted genes have similar therapeutic effects, we suggested these two drugs can be used for treating cancers. In fact, it has been reported that Floxuridine was recommended for patients suffering from cancer^21^, and the drug Lapatinib has been used in combination with Capecitabine for women with HER2-positive breast cancer^22^. In summary, the drug resistome from GEAR can help predict new indications for old drugs.

### Resistome based drug combinations

In clinic, the combinatorial therapies have been widely used to prevent the emergence of drug resistance^23^, where one agent may be used for inhibiting the resistant genes that confer resistance to the main drugs so that drug effects can be achieved. Therefore, it is possible to predict candidate drug combinations based on drug resistome. For example, in GEAR, BCL2 was recorded to be associated with resistance to Cisplatin, a drug widely used for cancer, where the cancer cells with expression of BCL2 were reported to be more significantly resistant to Cisplatin^24^. On the other hand, BCL2 is also a target protein of Paclitaxel, where BCL2 will be down-regulated and induced to be phosphorylated by Paclitaxel^25^. The combinatorial chemotherapy of Cisplain and Paclitaxel has been proved effective for Cisplatin-resistant human epidermoid carcinoma cell line by inducing apoptosis with the phosphorylation of BCL2^26^. In GEAR, ERBB2 has been annotated to enhance cell proliferation and prompt resistance of breast cancer to Letrozole, a drug is used for the treatment of hormonally-responsive breast cancer after surgery. As a tyrosine kinase inhibitor, Lapatibinb is able to suppress the expression of ERBB2. In fact, the combination between Letrozole and Lapatinib has been reported to increase the survival of patients with metastatic breast cancer^27^. Beyond recovering known drug combinations, the resistome from GEAR can help identify potential drug combinations for further investigation. For instance, in GEAR, the gene ABCB1 was annotated to be the resistant gene of Doxorubicin, which was a candidate transporter that efflux Doxorubicin and the upregulation of ABCB1 will acquire resistance to Doxorubicin. Recently, it has been found that the drug Imatinib can prevent acquired resistance of cancer cells to Doxorubicin by inhibiting the expression of ABCB1^28^. Thus, these two drugs may be used together to enhance the efficacy of Doxorubicin.

The cases shown above clearly demonstrated that the drug resistome in GEAR is really useful for narrowing down candidate drug combinations, and can be used with other information for identifying potential combinatorial therapies^29^.

## Conclusion

We introduce GEAR (A database of **G**enomic **E**lements **A**ssociated with drug **R**esistance), which provides comprehensive information about genetic factors causing human drug resistance, including variants in genes and miRNAs. We believe this valuable resource can help researchers to investigate the genetics underlying drug resistance, and help understand the molecular mechanisms underlying drug resistance. In addition, the drug resistome can help predict new indications of old drugs and potential drug combinations.

## Materials and Methods

### Data resource

We considered 121,870 published papers from MEDLINE until January 2014, where each paper has the MeSH Heading of ‘drug resistance’. All human approved drugs were retrieved from DrugBank (version 4.0)^30^, and human genes and miRNAs were respectively extracted from Entrez gene^31^ and miRBase^32^. Moreover, single-nucleotide polymorphisms (SNPs) from dbSNP database^33^ were also taken into account here. For each drug, all possible synonyms from PubChem^34^ Compound were adopted as its drug names. For genes, gene symbols as well as all their possible synonyms from HUGO Gene Nomenclature Committee (HGNC)^35^, Entrez and Uniprot^36^ were searched against MEDLINE. The miRNAs from miRBase and the SNPs from dbSNP were also used to query the MEDLINE.

### Paper parsing

For each paper, only the title and abstract were considered in this work. The associations between drugs and genes were derived based on their co-occurrence. If a drug and a gene co-occur in the same sentence in the abstract or title of one published paper, they are possibly related to each other. Furthermore, if the key word resistance/resistant/chemoresistance occurs around the drug name in the same sentence in which the gene also occurs, we supposed this gene is possibly responsible for the resistance to the drug. It is the same for miRNAs and SNPs. During paper parsing, common and stop word dictionary from STRING^37^ group were excluded from consideration in the name recognition.

Considering possible false positives in text mining, all associations between drug resistance and genomic elements (i.e. genes/miRNAs/SNPs) were further manually curated. As a result, 1631 associations between 201 drugs and 758 genes, 106 associations between 29 drugs and 66 miRNAs, and 44 associations between 17 drugs and 22 SNPs were kept and deposited into GEAR.

### Event ranking

The initial associations between drug resistance and genomic elements were extracted from MEDLINE with text mining, which results in 2387 associations. Then each drug and resistant genomic element association was manually checked by reading the sentences and references describing the resistance event. Consequently, 1781 associations were kept for further analysis. With the drug resistance events extracted above, it is possible that multiple drugs are associated with the same gene or a set of genes are reported to be related to the resistance of the same drug. Therefore, it is necessary to rank those events so that more potential events can be picked. To facilitate investigating associations between drugs and genes/miRNAs/SNPs, all associations were ranked based on the probability of their co-occurrence in MEDLINE with mutual information. For example, for drug *d* and gene *g*, the mutual information *MI(d, g*) between them can be calculated as follows.


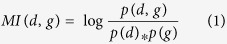


where *p(d, g*) is the probability of *d* and *g* co-occurring in the same paper, *p(d*) is the probability of *d* occurring in one paper, and the same for *p(g*). With the mutual information available, the associations with higher mutual information will be ranked top and therefore considered more confident. Note that the mutual information was only used as a proximity to confidence since we cannot say those resistant events with few publication support are false.

## Additional Information

**How to cite this article**: Wang, Y.Y. *et al*. GEAR: A database of Genomic Elements Associated with drug Resistance. *Sci. Rep.* **7**, 44085; doi: 10.1038/srep44085 (2017).

**Publisher's note:** Springer Nature remains neutral with regard to jurisdictional claims in published maps and institutional affiliations.

## Supplementary Material

Supplementary Table I

Supplementary Table II

## Figures and Tables

**Figure 1:**
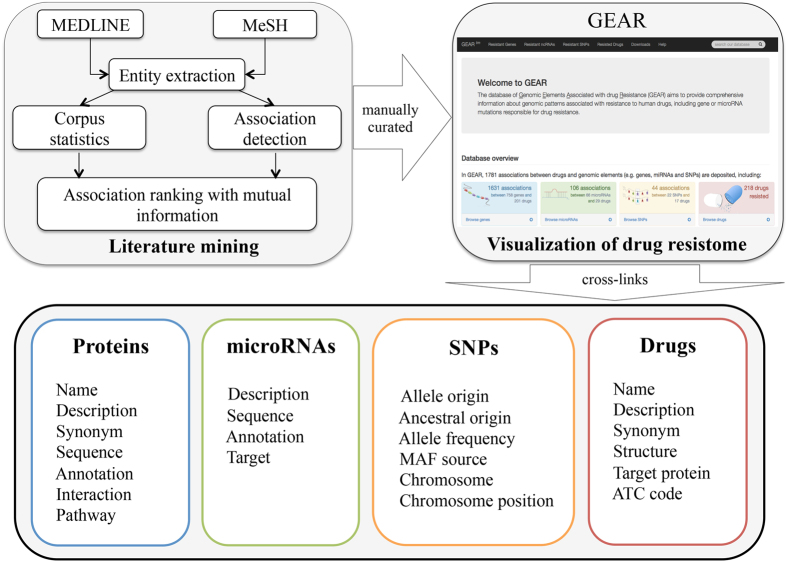
Schematic content of GEAR.

**Table 1:** The distribution of drugs according to their therapeutic categories.

Drug category	Number of drugs	Number of resistant genes
Antineoplastic and immunomodulating agents (L)	60	635
Antiinfectives for systemic use (J)	41	73
Dermatologicals (D)	22	78
Sensory organs (S)	19	60
Alimentary tract and metabolism (A)	15	51
Cardiovascular systems (C)	11	39
Genito-urinary system an sex hormones (G)	10	24
Musculo-skeletal system (M)	10	23
Antiparasitic products, insecticides and repellents (P)	8	16
Nervous system (N)	7	11
Various (V)	7	27
Respiratory system (R)	6	31
Systemic hormonal preparations, excluding sex hormones and insulins (H)	4	25
Blood and blood forming organs (B)	3	6
Not available (NA)	48	63

The dominant drug category was adopted for each drug, where the first level of the Anatomical Therapeutic Chemical (ATC) classification system was used as the therapeutic category.
